# Diagnostic Workup and Outcome in Patients with Profound Hyponatremia

**DOI:** 10.3390/jcm12103567

**Published:** 2023-05-19

**Authors:** Johann Isaak, Maria Boesing, Laura Potasso, Christoph Lenherr, Giorgia Luethi-Corridori, Joerg D. Leuppi, Anne B. Leuppi-Taegtmeyer

**Affiliations:** 1 University Centre of Internal Medicine, Cantonal Hospital Baselland, Rheinstrasse 26, 4410 Liestal, Switzerland; 2 Medical Faculty, University of Basel, Klingelbergstrasse 61, 4056 Basel, Switzerland; 3 Departments of Endocrinology, Diabetology and Metabolism, University Hospital Basel, Petersgraben 4, 4031 Basel, Switzerland; 4 Department of Clinical Nephrology, Cantonal Hospital of Baselland, Rheinstrasse 26, 4410 Liestal, Switzerland; 5 Department of Patient Safety, University Hospital Basel, Petersgraben 4, 4031 Basel, Switzerland

**Keywords:** sodium, audit, guidelines, rehospitalisation, survival, mortality

## Abstract

Hyponatremia is the most common electrolyte disorder. A proper diagnosis is important for its successful management, especially in profound hyponatremia. The European hyponatremia guidelines point at sodium and osmolality measurement in plasma and urine, and the clinical evaluation of volume status as the minimum diagnostic workup for the diagnosis of hyponatremia. We aimed to determine compliance with guidelines and to investigate possible associations with patient outcomes. In this retrospective study, we analysed the management of 263 patients hospitalised with profound hyponatremia at a Swiss teaching hospital between October 2019 and March 2021. We compared patients with a complete minimum diagnostic workup (D-Group) to patients without (N-Group). A minimum diagnostic workup was performed in 65.5% of patients and 13.7% did not receive any treatment for hyponatremia or an underlying cause. The twelve-month survival did not show statistically significant differences between the groups (HR 1.1, 95%-CI: 0.58–2.12, *p*-value 0.680). The chance of receiving treatment for hyponatremia was higher in the D-group vs. N-Group (91.9% vs. 75.8%, *p*-value < 0.001). A multivariate analysis showed significantly better survival for treated patients compared to not treated (HR 0.37, 95%-CI: 0.17–0.78, *p*-value 0.009). More efforts should be made to ensure treatment of profound hyponatremia in hospitalised patients.

## 1. Introduction

Hyponatremia, defined as a decrease in plasma sodium concentration <135 mmol/L, is the most common disorder of electrolyte and water balance, affecting up to 30% of hospitalised patients [1]. Profound hyponatremia (plasma sodium < 125 mmol/L) is less common with a prevalence of 0.15–2.5% [2,3,4]. Hyponatremia is associated with increased morbidity and mortality [5,6,7], as shown in patients with heart failure and myocardial infarction [8,9,10,11], liver cirrhosis [12,13], pneumonia [14], chronic obstructive pulmonary disease [15] and stroke [16]. Moreover, studies showed an association between hyponatremia and increased risk of falls, fractures and osteoporosis [17,18]. 

Profound hyponatremia is associated with an increased rate of rehospitalisation and mortality, may often require admission to an intensive care unit and has been identified as an independent risk factor for poor prognosis [19,20]. The need for immediate treatment in acute, symptomatic hyponatremia, which can be severe and potentially life threatening, is well-recognised [19,21,22,23,24]. In contrast, aggressive overcorrection in patients with chronic hyponatremia can cause osmotic demyelination with potentially irreversible neurologic deficits [22,25,26,27]. The cause and treatment of hyponatremia differ from case to case [3,22,28,29]. A proper diagnosis of hyponatremia is important for its successful management, but identifying the underlying cause remains challenging in the hospital setting [5,30,31]. Deficits in the adequate management of patients hospitalised with profound hyponatremia have been identified by previous studies [4,29,32,33,34,35]. 

We audited the management of hospitalised patients with profound hyponatremia at the Cantonal Hospital Baselland (KSBL) in Switzerland for diagnostic workup and therapeutic management, adherence to current guidelines and patient’s outcome. The European hyponatremia guidelines recommend sodium and osmolality measurement in plasma and urine, and a clinical evaluation of the volume status as the minimum workup for diagnosing hyponatremia. Depending on the findings and the resulting diagnosis, treatment differs according to the underlying cause [3].

The aim of our study was to determine compliance to guidelines in terms of diagnostic workup and treatment of profound hyponatremia. Moreover, we aimed to investigate possible associations with clinical outcome in terms of rehospitalisation, discharge destination and mortality in patients with and without a minimum diagnostic workup as well as in patients receiving treatment of their profound hyponatremia or not.

## 2. Materials and Methods

### 2.1. Study Design and Patient Selection

We performed a retrospective, observational hospital record study at the three sites of the Cantonal Hospital Baselland (KSBL) in Switzerland (Bruderholz, Laufen and Liestal). KSBL has a total of 511 beds, serves a catchment population of 140,000 inhabitants and had approximately 16,650 hospitalisations in 2020 [36].

A laboratory database search was conducted for patients with plasma sodium <125 mmol/L in the 18-months period between October 2019 and March 2021 using the i-engine HE^®^ program. Adult, hospitalised patients from all disciplines were included in the study (general medicine, geriatric medicine and surgical departments). The chosen time period was five years after the 2014 hyponatremia clinical practice guidelines and before the initiation of the “Hyponatremia Intervention Trial” (HIT) [37]. The HIT is an international, randomised, controlled, multicentre trial to study the effects of targeted correction of plasma sodium versus standard of care, which started at KSBL in April 2021 and could have been a bias to our audit.

The threshold of 125 mmol/L was selected because, according to European guidelines, it defines profound hyponatremia and several studies have shown an association with worse outcomes [19,23,34,38]. Whenever a patient was hospitalised more than once during this period, only the data of the first hospitalisation were assessed as index hospitalisation. 

**Figure 1:**
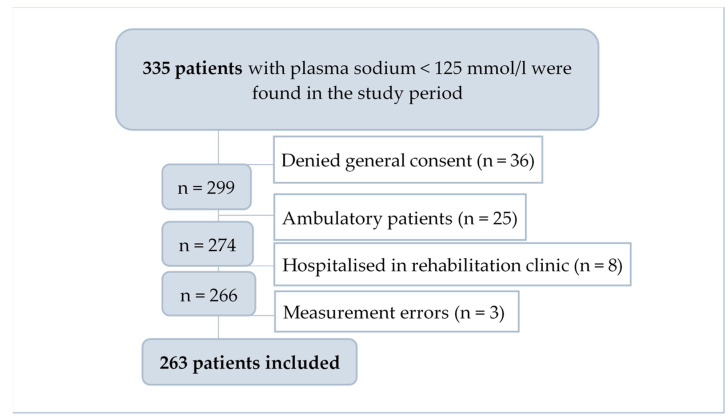
Flowchart diagram for patient selection process.

Sodium analysis was performed in venous blood, either using an accredited method with ion selective electrodes of the Roche Cobas Pure at Bruderholz, Roche Cobas Integra 400 at Laufen and Roche Cobas 8000 at Liestal or with the bedside blood gas analyses from Radiometer ABL machines. Of the 335 initially screened patients, 36 denied general research consent, 25 were ambulatory patients, eight were hospitalised in the rehabilitation department and three were found to be measurement errors. A total of 263 patients were enrolled in the study (Figure 1). 

The study protocol was reviewed and approved by the ethics committee of northwest and central Switzerland (ENKZ BASEC Project-ID 2022-02004). Only patients who had not denied general informed consent for anonymous use of their health-related data for research purposes were included in the study. 

### 2.2. Data Collection

Data collection was performed manually, searching all available electronic patient records, and managed using Research Electronic Data Capture (REDCap^®^). REDCap^®^ is a secure, web-based software platform designed to support data capture for research studies [39,40]. The hyponatremia clinical practice guidelines established by the European Societies of Endocrinology, Intensive Care Medicine and the European Renal and Dialysis & Transplant associations in 2014 were used as the state-of-the-art comparator for the diagnostic approach [3]. We analysed the patients’ data for baseline characteristics, installed medication at presentation and comorbidities. We stratified the patients according to their Charlson comorbidity index [41,42]. We registered if hyponatremia was the reason for hospitalisation or not, diagnostic workup, initial treatment setting and treatment received, plasma sodium correction rate, occurrence of complications, successful correction of plasma sodium prior to discharge or not, length of hospital stay, discharge destination, rehospitalisation and mortality. 

In order to investigate the effect of diagnostic workup on patient outcomes, we assigned patients to one of two groups: the D-group included patients who received the minimum required diagnostic workup consisting of the measurement of plasma and urine osmolality, plasma and urine sodium, and the assessment of the patient’s body volume status. The N-group consisted of patients who had not been investigated with minimum diagnostic workup. We scanned patient files for prescribed and received treatment independent of the presence or absence of a formulated treatment plan in the documentation.

We also compared whether the diagnosed aetiology and treatment were congruent according to treatment recommendations [3]. Finally, the two groups were compared regarding rehospitalisation within one year, in-hospital mortality and one-year mortality. We also compared the group of patients who received treatment for profound hyponatremia with those who did not receive any treatment for it.

### 2.3. Statistical Analysis

The descriptive statistical analysis was performed with REDCap^®^ and the comparative and inferential statistical analysis was performed with R version 4.0.3 [43]. The Mann–Whitney U test was used for continuous variables and the Chi Square test was used for categorical variables to determine group-wise differences. Time-to-event data (time to death, time to rehospitalisation) was analysed with Cox-proportional hazards models, adjusted for plasma sodium at diagnosis, polypharmacy and the Charlson comorbidity index. We confirmed the proportional hazards assumption using scaled Schoenfeld residuals with the function “co.zph()” in R. Kaplan–Meier curves were plotted with GraphPad Prism version 9. GraphPad Prism is a statistical analysis software that combines scientific graphing, comprehensive curve fitting (nonlinear regression), understandable statistics and data organisation [44].

## 3. Results

### 3.1. Clinical Characteristics

The overall prevalence of profound hyponatremia in hospitalised patients in our study period of 18 months was 1.34%. The baseline characteristics of the 263 patients with a median age of 77 years and a female predominance of 65.4% are shown in Table 1, overall and divided by group according to the diagnostic approach.

**Figure 2:**
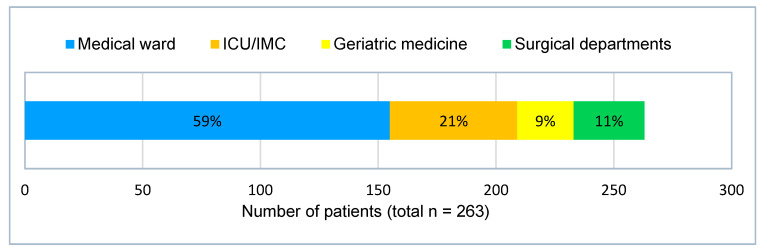
Initial treatment setting after diagnosis of profound hyponatremia, ICU = intensive care unit, IMC = intermediate care unit.

Figure 2 shows the patients’ initial treatment settings after the initial diagnosis of profound hyponatremia. One hundred fifty-five patients (59%) were treated in the medical ward. Twenty-four patients (9%) were treated in the department for geriatric medicine and 30 patients (11%) were treated in surgical departments. Fifty-four patients (21%) were initially admitted to the intensive care unit (ICU) or intermediate care unit (IMC). Of those, hyponatremia was the reason for admission to a high dependency department in 16 cases (30%). 

### 3.2. Diagnostic Workup

As suggested by the clinical practice guidelines [3], the measurement or calculation of plasma osmolality, and measurement of urine osmolality and sodium, and clinical assessment of the volume status are included in the minimum diagnostic workup for the diagnosis of hyponatremia. In our study, this was performed in 172 patients (65.5%) (Table 2). In some patients, urine osmolality was measured but not urine sodium and vice versa. Any remarks on the clinical volume status (e.g., absence or presence of pitting oedema, absence or presence of jugular vein congestion, etc.) were documented in 233 patients (88.6%).

**Figure 3:**
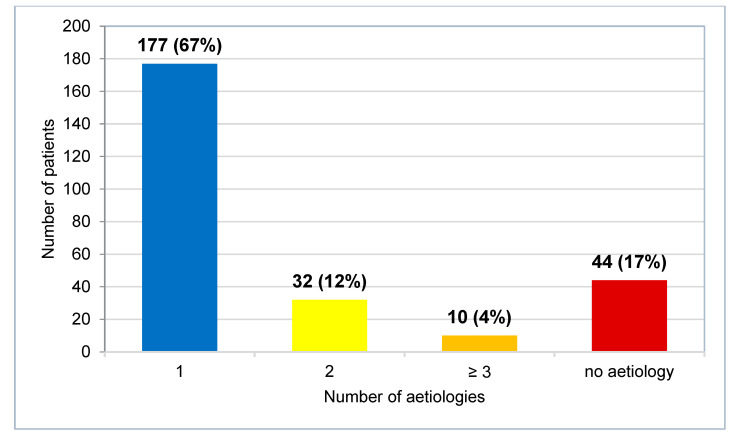
Number of patients grouped according to number of documented aetiologies for profound hyponatremia.

In 44 cases (17%), clinicians did not determine any aetiology. In 42 cases (16%), clinicians suggested multiple aetiologies for hyponatremia or diagnosed multiple causes (see Table S1). In 177 cases (67%), clinicians determined a single cause (Figure 3). 

**Figure 4:**
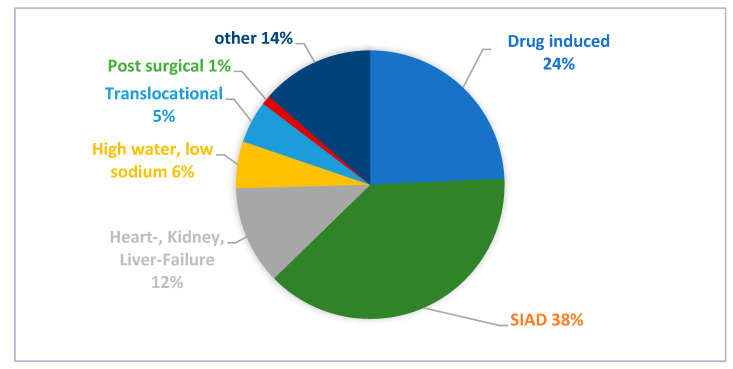
Distribution of documented aetiologies when clinicians suggested a single cause. SIAD = syndrome of inappropriate antidiuresis.

The distribution of aetiologies in the 177 patients for whom clinicians determined a single cause of hyponatremia is presented in Figure 4, with syndrome of inappropriate antidiuresis (SIAD) identified as the leading diagnosis, suggested as a possible cause in 68 patients (38%). The diagnostic criteria for SIAD according to Schwartz & Barttner [45] were not satisfied in most cases (80%) due to a lack of documented clinical volume status or failure to exclude adrenal, thyroid, pituitary and renal insufficiency. Forty-eight patients (27%) with a single determined aetiology had not completed the minimum diagnostic workup.

### 3.3. Treatment

**Figure 5:**
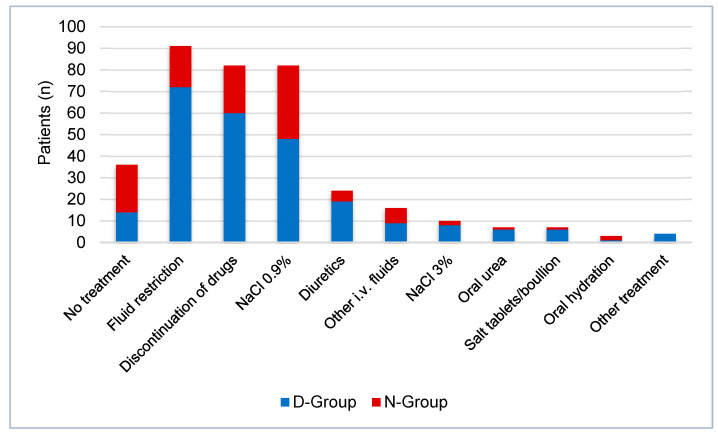
Treatment of hyponatremia in patients with plasma sodium < 125 mmol/L by group, D-Group: minimum diagnostic workup complete, N-Group: incomplete minimum diagnostic workup.

Thirty-six patients (14%) with profound hyponatremia did not receive any treatment for hyponatremia or its underlying cause. An intravenous infusion of 3% NaCl was administered in 10 patients (4%), seven of which suffered acute or symptomatic hyponatremia. Vaptans or demeclocycline were not administered to any patients in our study population. Figure 5 shows the different treatments given. According to identified aetiology, 141 patients (44%) received congruent treatment and 53 patients (20%) did not receive treatment fitting the suggested aetiology. In 69 cases (26%), treatment categorisation in adherence to guidelines was not applicable due to missing aetiology, treatment or both.

In 23 cases, patients with initially suspected SIAD were treated with 0.9% sodium chloride solution, which in four cases led to a further decline in plasma sodium. One of these patients was subsequently treated with hypertonic 3% sodium chloride solution, which led to the correction of serum sodium. The average sodium correction rate was 3 mmol/day in the first two days. Patients in the D group were more likely to receive treatment for hyponatremia or an underlying cause than patients in the N group (91.9% vs. 75.8%, *p*-value < 0.001).

### 3.4. Outcome

Table 3 shows the outcome overall and stratified by the diagnostic workup group. In 43 cases (16.3%), hyponatremia was the main diagnosis. Thirty-seven of these cases (86.0%) had complete minimum diagnostic workup. In 24 cases (9.1%), hyponatremia was not mentioned in the discharge report at all and therefore not coded in the Diagnosis Related Groups (DRG), on which the hospital reimbursement system is based. Eight of these cases did have a complete minimum diagnostic workup (33.3%).

The proportion of patients with persistency of profound hyponatremia at discharge was the same in both D- und N-Group (8.1% vs. 8.8%, *p*-value 0.126). In 12 cases (4.6%), plasma sodium at discharge was the same or worse than at diagnosis. The distribution of discharge destination differed between the two groups: in the D-Group, more patients could be discharged home (73.2% vs. 65.9%, *p* = 0.214) whereas in the N-Group more patients needed a transfer to another hospital (5.8% vs. 7.7%, *p* = 0.556).

**Figure 6:**
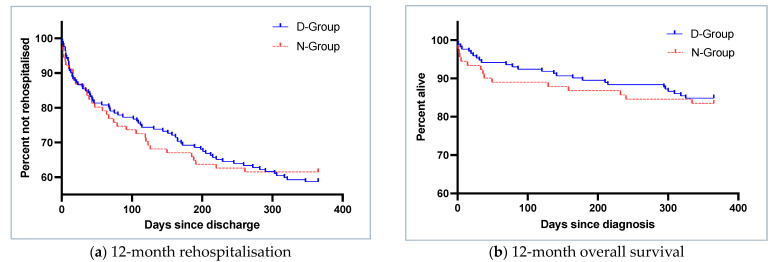
Kaplan–Meier curves for (**a**) 12-month rehospitalisation by group (log rank, *p*-value 0.82) and (**b**) 12-month overall survival by diagnostic group (log-rank, *p*-value 0.68), D-Group: minimum diagnostic workup complete, N-Group: incomplete minimum diagnostic workup.

Rehospitalisation within 12 months was more frequent in the D-Group, but differences were not statistically significant (42.5% vs. 41.2%, *p* = 0.839) (Figure 6a). 

Cox proportional hazards regression, corrected for plasma sodium at diagnosis, polypharmacy and Charlson comorbidity index, did not show statistically significant differences in 12-month overall survival (hazard ratio (HR) D-Group—vs. N-Group 1.1, 95%-confidence interval (CI): 0.578–2.124, *p*-value 0.680) (Figure 6b). 

**Figure 7:**
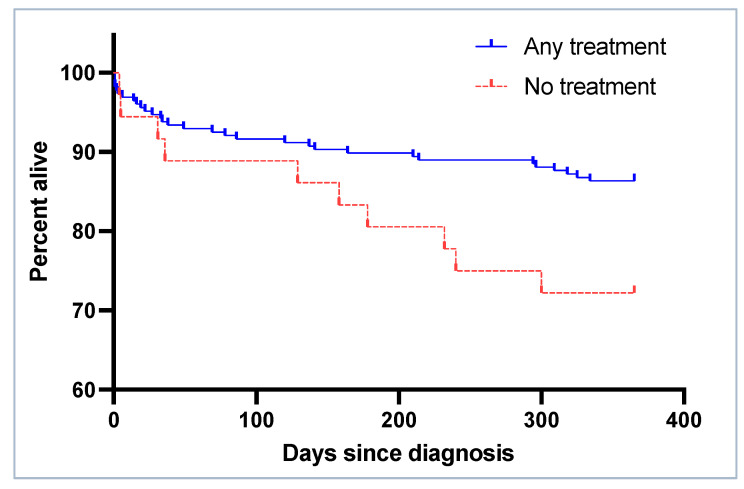
Kaplan–Meier curves for 12-month survival by treatment (log rank *p*-value 0.03).

The twelve-month overall survival was significantly better for patients receiving treatment for the hyponatremia as compared to those who did not (Figure 7). Cox proportional hazards regression corrected for plasma sodium at diagnosis, polypharmacy and Charlson comorbidity index revealed an association between treatment of hyponatremia and the survival rate (HR any treatment vs. no treatment 0.366, 95%-CI: 0.172–0.779, *p*-value: 0.009). The chance of receiving treatment was higher in the D-Group than in the N-Group (91.9% vs. 75.8%, *p*-value 0.030).

Table 4 compares the baseline characteristics of patients who received treatment and those who did not. In patients who received treatment, hyponatremia was mentioned in the discharge report in 95% of cases. In patients who were not treated, this figure was 64% (*p*-value < 0.001). 

## 4. Discussion

Our real-life study of profound hyponatremia in a Swiss teaching hospital has four main findings. First, the compliance with the guidelines for the diagnosis of hyponatremia in hospitalised patients [3] was moderately low, with the minimum required diagnostic workup having been performed in 65.5% of patients. Second, there was no difference in terms of rehospitalisation or survival within 12-months between patients with minimum diagnostic workup (D-Group) and those without (N-Group). Third, patients in the D-Group were more likely to receive treatment for hyponatremia. Fourth, this treatment was associated with a significantly better 12-month overall survival. 

Hyponatremia is a complex disorder with a wide range of possible underlying causes [3,22]. The application of a diagnostic workup for the differentiation of hyponatremia changes from case to case [3,5,22,28]. On the other hand, an adequate determination of the type and cause of the hyponatremia is essential for patient treatment in order to avoid the further deterioration of plasma sodium levels and possible severe consequences for the patients [32]. Despite being the most common electrolyte disorder, hyponatremia remains underdiagnosed [35]. Profound hyponatremia is less common, but as it could be associated with worse outcome, it is particularly relevant [2,5,8,31,38]. In our study, the prevalence of profound hyponatremia was 1.34%, which is in line with previous studies, where the prevalence ranged from 0.15 to 2.5% [1,2,4]. The length of hospital stay was longer in the D-Group. One possible explanation could be that the diagnostic workup, which led to the treatment of patients, took more time. Further decline in plasma sodium during the hospitalisation happened more often in the N-Group and overcorrection happened less often in the N-Group, which can both be explained by the lower rate of treatment in the N-Group. 

The diagnostic workup of profound hyponatremia in other studies was also found to be low. It has been reported previously that only 31–33% of patients with profound hyponatremia underwent serum-osmolality, urine-osmolality and urine sodium measurement [4,31], although it must be noted that the threshold for profound hyponatremia in these two studies was <120 mmol/L. One study reported that only 10–27% of patients with a plasma sodium <125 mmol/L had their plasma osmolality and urinary indices checked [32]. Compared to these studies, in our audit, the frequency of diagnostic laboratory workup was higher (72.2%). One could argue that plasma osmolality can be estimated using the formula 2 [Sodium] + [Glucose] + [Urea] [46]. While plasma glucose and urea were measured in 95.4%, it did not lead to the testing of urine indices. If we assume that clinicians always used the formula to estimate plasma osmolality, complete minimum diagnostic laboratory workup would have been performed in 76.8% instead of 72.2% for laboratory workup alone. In our study, the clinical volume status was assessed and documented in 88.6% of the patients. In audits of other hospitals, the evaluation of clinical volume status of patients with plasma sodium <120 mmol/L was performed and documented in 40–65% of cases [4,31]. However, previous studies have shown that determination of the correct clinical volume status can be challenging [47]. 

The minimum diagnostic workup in our study was not significantly associated with a better outcome regarding in-hospital mortality. This could be partly due to the fact that our patients had a lower in-hospital mortality (4.2%) compared to other studies where in-hospital mortality ranged from 7–50% [4,31]. Further, our study did not show statistically significant differences of rehospitalisation within 12 months between the two groups. This could be due to the relatively small sample size, which might not detect a possible effect. Another possible explanation could be that diagnostic workup and treatment were not performed in patients receiving palliative care. However, in our study population, none of the patients were initially treated with terminal, palliative care. On the other hand, we showed that patients who were treated for hyponatremia had a significantly better 12-month overall survival than those who were not treated and the chance of being treated was significantly higher when a minimal diagnostic workup was performed. Associations between active management and improved mortality of profound hyponatremia have been reported before [48]. In our study, we observed that patients who did not receive treatment did not have poorer health (as indicated by the Charlson comorbidity index, as shown in Table 4) or substance abuse, and were not more likely to be receiving hyponatremia-triggering medication that could not easily be withdrawn (for example antipsychotic or antiseizure drugs).

Another important point was to compare if the treatment of profound hyponatremia was congruent with the suggested aetiology of hyponatremia. In 20.2%, treatment did not fit the suggested aetiology. In some cases, patients with initially suspected SIAD were treated with 0.9% sodium chloride solution, which lead to a further decline in plasma sodium. This shows that the aetiologies and their treatment are not always well understood and there is need for improvement. We did observe that in 24% of patients without minimum diagnostic workup and in 36% of patients without treatment, hyponatremia was not mentioned at all in the discharge report. We therefore conclude that in several cases, clinicians failed to recognise the presence of profound hyponatremia or the need for its treatment.

Several improvements can be implemented to increase the guideline conformity and quality of hyponatremia management: a laboratory result showing profound hyponatremia should trigger testing of plasma osmolality and plasma glucose and urea, along with the testing of urine indices including urine osmolality and urine sodium. In addition, the assessment of the patient‘s clinical volume status could help in determining the correct aetiology and to start treatment accordingly. The diagnosis of a profound hyponatremia should also be better documented in the discharge report in order to reflect a proper diagnosis related groups (DRG) coding.

### Limitations

While the findings of this study reflect everyday real-life clinical practice at our institution, the study has a number of limitations, mainly related to its retrospective, observational design. Missing data such as volume status or aetiology were rated as not assessed, even though there was a possibility that they had been assessed, but not documented. Therefore, the quality of care could have been underestimated. Retrospectively, it was also not always possible to determine if a patient’s symptoms (such as vomiting, for example) were the cause or the consequence of hyponatremia. 

## 5. Conclusions

Our study highlights the importance of adhering to clinical practice guidelines for diagnosing and treating profound hyponatremia. In our study, we showed that adherence to clinical practice guidelines and the application of diagnostic workup should be improved. Patients whose workup adhered to clinical practice guidelines did not show significantly better 12-month overall survival or lower rehospitalisation rates than patients whose workup was not adherent. However, patients with completed minimal diagnostic workup were more likely to receive treatment for hyponatremia, and treatment was associated with a significantly better 12-month overall survival than no treatment. Concerted efforts should therefore be made to ensure treatment of profound hyponatremia at our institution. Large, prospective studies are needed to confirm these findings.

## Figures and Tables

**Table 1:** Baseline characteristics of patients with plasma sodium < 125 mmol/L overall and by group.

	Overall (n = 263)	D-Group (n = 172)	N-Group (n = 91)	*p*-Value
Age (years), median, IQR	77, 67–85	77, 68–85	77, 66.5–85	0.782
Sex female, n (%)	172 (65.4)	106 (61.6)	66 (72.5)	0.077
BMI (kg/m^2^), median, IQR	24.4, 21.8–28.1	25.1, 21.8–28.2	24.0, 21.8–27.6	0.429
Nursing home residents, n (%)	32 (12.2)	22 (12.8)	10 (11.0)	0.671
P-Na at diagnosis (mmol/L), median, IQR	122, 119–124	121, 118–123	123, 121–124	<0.001
Charlson comorbidity index 0–4, n (%)	126 (47.9)	80 (46.5)	46 (50.6)	0.533
Charlson comorbidity index 5–7, n (%)	137 (52.1)	92 (53.5)	45 (49.5)	0.533
Comorbidities				
Cardiovascular disease, n (%)	203 (77.2)	133 (77.3)	70 (76.9)	0.941
Diabetes mellitus, n (%)	59 (22.4)	35 (20.35)	24 (26.4)	0.265
Mental health disorder, n (%)	44 (16.7)	25 (14.5)	19 (20.9)	0.190
Dementia, n (%)	39 (14.8)	24 (14.0)	15 (16.5)	0.583
COPD, n (%)	37 (14.1)	20 (11.6)	17 (18.7)	0.118
Chronic pain syndrome, n (%)	36 (13.7)	25 (14.5)	11 (12.1)	0.583
Substance addiction, n (%)	23 (8.8)	21 (12.2)	2 (2.2)	0.006
Severe CKD, n (%)	23 (8.8)	13 (7.6)	10 (11.0)	0.349
Malignant disease, n (%)	30 (11.4)	19 (7.2)	11 (4.2)	0.800
Any regular medication, n (%)	243 (92.4)	161 (93.6)	82 (90.1)	0.309
Polypharmacy ≥ 5, n (%)	190 (72.4)	123 (71.5)	67 (73.6)	0.716
Patients with hyponatremia associated medications, n (%)	224 (85.2)	149 (86.6)	75 (82.4)	0.361
Diuretics, n (%)	129 (49.1)	85 (49.4)	44 (48.4)	0.869
Thiazides and thiazide-like, n (%)	81 (30.8)	56 (32.6)	25 (27.5)	0.395
MRA, n (%)	24 (9.1)	14 (8.1)	10 (11.0)	0.445
RAAS-inhibitors, n (%)	158 (60.1)	104 (60.5)	54 (59.3)	0.859
ACE-inhibitors, n (%)	67 (25.5)	50 (29.1)	17 (18.7)	0.066
ARBs, n (%)	91 (34.6)	54 (31.4)	37 (40.7)	0.133
Antidepressants, n (%)	41 (15.6)	24 (14.0)	17 (18.7)	0.315
Tricyclic/tetracyclic, n (%)	20 (7.6)	12 (7.0)	8 (8.8)	0.597
SSRI/SSNRI, n (%)	18 (6.8)	12 (7.0)	6 (6.6)	0.907
SARI/MAO-I, n (%)	10 (3.8)	4 (2.3)	6 (6.6)	0.085
Antiseizure drugs, n (%)	37 (14.1)	25 (14.5)	12 (13.2)	0.765
Antipsychotic drugs, n (%)	24 (9.1)	10 (5.8)	14 (15.4)	0.010
Benzodiazepines, n (%)	17 (6.46)	8 (4.7)	9 (9.9)	0.100

Abbreviations: ACE = angiotensin converting enzyme, ARBs = angiotensin receptor blockers (Sartans), BMI = body mass index, COPD = chronic obstructive pulmonary disease, IQR = interquartile range, MAO-I = monoamine oxidase inhibitors, MRA = mineralocorticoid receptor antagonist, P-Na = plasma sodium, RAAS = renin–angiotensin–aldosterone system, SARI = serotonin antagonist and reuptake inhibitors, SSNRI = selective serotonin noradrenalin reuptake inhibitors, SSRI = selective serotonin reuptake inhibitors. D-Group: minimum diagnostic workup complete, N-Group: incomplete minimum diagnostic workup.

**Table 2:** Diagnostic workup of patients with plasma sodium < 125 mmol/L overall.

	Total (n = 263)
1. Plasma diagnostic workup	
Plasma osmolality measured, n (%)	213 (81.0)
Plasma glucose measured, n (%)	252 (95.8)
Plasma urea measured, n (%)	258 (98.1)
2. Urine osmolality measured, n (%)	215 (81.7)
3. Urine sodium measured, n (%)	218 (82.9)
*Laboratory workup performed ^1^, n (%)*	*190 (72.2)*
4. Volume status documented in medical records, n (%)	233 (88.6)
*Complete minimal diagnostic workup performed ^2^, n (%)*	*172 (65.5)*
5. Aetiology documented in discharge report, n (%)	219 (83.3)
6. Formulated treatment plan in discharge report, n (%)	194 (73.8)

^1^ consisting of sodium and osmolality in plasma and urine. ^2^ consisting of sodium and osmolality in plasma and urine, and clinical volume status.

**Table 3:** Outcome for patients hospitalised with plasma sodium < 125 mmol/L, overall and by group.

	Overall (n = 263)	D-Group (n = 172)	N-Group (n = 91)	*p*-Value
Hyponatremia as main diagnosis, n (%)	43 (16.3)	37 (14.1)	6 (2.3)	0.007
Hyponatremia not mentioned in discharge report, n (%)	24 (9.1)	8 (3.0)	16 (6.1)	0.001
No treatment received for hyponatremia, n (%)	36 (13.7)	14 (8.1)	22 (24.2)	<0.001
Complications, n (%)	47 (17.9)	31 (18.0)	16 (17.6)	0.929
Further decline in plasma sodium, n (%)	27 (10.3)	16 (9.3)	11 (12.1)	0.479
Overcorrection ^1^, n (%)	18 (6.8)	14 (8.1)	4 (4.4)	0.253
Subsequent transfer to ICU/IMC, n (%)	2 (0.8)	1 (0.6)	1 (1.1)	0.646
ODS (osmotic demyelination syndrome), n (%)	0 (0.0)	0 (0.0)	0 (0.0)	N/A
Brain stem herniation, n (%)	0 (0.0)	0 (0.0)	0 (0.0)	N/A
P-Na < 125 mmol/L prior to discharge, n (%)	22 (8.4)	14 (8.1)	8 (8.8)	0.126
Length of hospital stay (days), median, IQR	9, 5–14	9, 6–14	8, 4–14	0.151
Discharge destination				
Home, n (%)	186 (70.7)	126 (73.3)	60 (65.9)	0.214
First ever in nursing home, n (%)	1 (0.4)	0 (0.0)	1 (1.1)	0.168
Rehabilitation clinic, n (%)	48 (18.3)	31 (18.0)	17 (18.7)	0.895
Other hospital, n (%)	17 (6.5)	10 (5.8)	7 (7.7)	0.556
Rehospitalisation within 12 months ^2^, n (%)	106 (42.1)	71 (42.5)	35 (41.2)	0.839
Hyponatremia at rehospitalisation, n (%)	72 (67.9)	52 (73.2)	20 (57.1)	0.095
Hyponatremia main diagnosis at rehospitalisation, n (%)	10 (13.9)	6 (8.5)	4 (11.4)	0.352
All cause death within 12 months, n (%)	41 (15.6)	26 (12.8)	15 (16.5)	0.876
All cause in-hospital death, n (%)	11 (4.2)	5 (2.9)	6 (6.6)	0.155

^1^ defined as sodium correction rate >10 mmol/L in 24 h or >18 mmol/L in 48 h after diagnosis, ICU = intensive care unit, IMC = intermediate care unit, ^2^ 11 patients died in index hospitalisation and could not be rehospitalised, P-Na = plasma sodium, D-Group: minimum diagnostic workup complete, N-Group: incomplete minimum diagnostic workup.

**Table 4:** Baseline characteristics of patients who were treated and those who were not.

	Any Treatment (n = 227)	No Treatment (n = 36)	*p*-Value
Age (years), median, IQR	78, 68–85	72, 60–82	0.044
Sex female, n (%)	151 (66.5)	21 (58.3)	0.441
BMI (kg/m^2^), median, IQR	24.9, 22.0–28.6	22.3, 21.0–26.0	0.018
Nursing home residents, n (%)	23 (12.8)	3 (8.3)	0.629
P-Na at diagnosis (mmol/L), median, IQR	122, 118.5–123.0	123.5, 122–124	<0.001
Charlson comorbidity index 0–4, n (%)	106 (46.7)	20 (55.6)	0.419
Charlson comorbidity index 5–7, n (%)	121 (53.3)	16 (44.4)	0.419
Comorbidities			
Cardiovascular disease, n (%)	177 (78.0)	26 (72.2)	0.582
Diabetes mellitus, n (%)	52 (22.9)	7 (19.4)	0.804
Mental health disorder, n (%)	39 (17.2)	5 (13.9)	0.802
Dementia, n (%)	32 (14.1)	7 (19.4)	0.558
COPD, n (%)	28 (12.3)	9 (25.0)	0.076
Chronic pain syndrome, n (%)	36 (15.9)	0 (0.0)	0.021
Substance addiction, n (%)	20 (8.8)	3 (8.3)	1.000
Severe CKD, n (%)	22 (9.7)	1 (2.8)	0.295
Malignant disease, n (%)	24 (10.6)	6 (16.7)	0.432
Any regular medication, n (%)	211 (93.0)	32 (88.9)	0.606
Polypharmacy ≥ 5, n (%)	167 (73.6)	23 (63.9)	0.315
Patients with hyponatremia associated medications, n (%)	198 (87.2)	26 (72.2)	0.036
Diuretics, n (%)	117 (51.5)	12 (33.3)	0.064
Thiazides and thiazide-like, n (%)	77 (33.9)	4 (11.1)	0.010
MRA, n (%)	18 (7.9)	6 (16.7)	0.168
RAAS-inhibitors, n (%)	139 (61.2)	19 (52.8)	0.436
ACE-inhibitors, n (%)	60 (26.4)	7 (19.4)	0.491
ARBs, n (%)	79 (34.8)	12 (33.3)	1.000
Antidepressants, n (%)	32 (14.1)	9 (25.0)	0.153
Tricyclic/tetracyclic, n (%)	15 (6.6)	5 (13.9)	0.233
SSRI/SSNRI, n (%)	15 (6.6)	3 (8.3)	0.980
SARI/MAO-I, n (%)	9 (4.0)	1 (2.8)	1.000
Antiseizure drugs, n (%)	31 (13.7)	6 (16.7)	0.822
Antipsychotic drugs, n (%)	23 (10.1)	1 (2.8)	0.266
Benzodiazepines, n (%)	14 (6.2)	3 (8.3)	0.900

Abbreviations: ACE = angiotensin converting enzyme, ARBs = angiotensin receptor blockers (Sartans), BMI = body mass index, COPD = chronic obstructive pulmonary disease, IQR = interquartile range, MAO-I = monoamine oxidase inhibitors, MRA = mineralocorticoid receptor antagonist, P-Na = plasma sodium, RAAS = renin–angiotensin–aldosterone system, SARI = serotonin antagonist and reuptake inhibitors, SSNRI = selective serotonin noradrenalin reuptake inhibitors, SSRI = selective serotonin reuptake inhibitors. D-Group: minimum diagnostic workup complete, N-Group: incomplete minimum diagnostic workup.

## Data Availability

The data presented in this study are available on reasonable request from the corresponding author. The data are not publicly available due to restrictions in data privacy.

## Supplementary Materials

The following supporting information can be downloaded at: https://www.mdpi.com/article/10.3390/jcm12103567/s1. The diagnoses among patients with multiple aetiologies for hyponatremia are shown in Table S1. All other data generated or analysed during this research are included in this article. Further inquiries can be directed to the corresponding author.
