# Supplementary material for: Diagnostic Workup and Outcome in Patients with Profound Hyponatremia
Source: J Clin Med. 2023 May 19;12(10):3567. doi: 10.3390/jcm12103567 (PMC10219478; doi:10.3390/jcm12103567)
Supplement: Supplementary file 1 [file jcm-12-03567-s001.zip › jcm-2348681-supplementary.pdf]

Table S1. Diagnoses among patients with multiple aetiologies for hyponatremia (n = 42).

Aetiology	Number of Patients
Drug-induced	26
SIAD	35
Heart, kidney, liver failure	7
High water, low sodium	11
Translocational	1
Post-surgical	0
Other[1]	16

[1] pseudohyponatremia, gastrointestinal sodium loss, hypovolemia not otherwise specified, central and primary adrenal insufficiency, cerebral salt wasting, hypothyroidism.
